# Antimicrobial susceptibility and genetic characteristics of *Neisseria gonorrhoeae* isolates from India, Pakistan and Bhutan in 2007–2011

**DOI:** 10.1186/1471-2334-13-35

**Published:** 2013-01-24

**Authors:** Sunil Sethi, Daniel Golparian, Manju Bala, Dorji Dorji, Muhammad Ibrahim, Kausar Jabeen, Magnus Unemo

**Affiliations:** 1 Department of Medical Microbiology, Post Graduate Institute of Medical Education and Research, Chandigarh, India; 2 WHO Collaborating Centre for Gonorrhoea and other STIs, National Reference Laboratory for Pathogenic Neisseria, Department of Laboratory Medicine, Clinical Microbiology, Örebro University Hospital, SE-701 85, Örebro, Sweden; 3 WHO GASP SEAR Regional Reference Laboratory, Apex Regional STD Teaching, Training & Research Centre, VMMC & Safdarjang Hospital, New Delhi, India; 4 JDW/NR Hospital, Thimphu, Bhutan; 5 The Aga Khan University, Karachi, Pakistan

**Keywords:** Gonorrhoea, Antimicrobial resistance, *penA*, Ceftriaxone, Southeast Asia, India, Bhutan, Pakistan

## Abstract

**Background:**

Knowledge on antimicrobial drug resistance and genetic characteristics of *Neisseria gonorrhoeae* isolates circulating in India, Pakistan, and Bhutan is sorely lacking. In this paper, we describe the prevalence of antimicrobial resistance and molecular characteristics of *N. gonorrhoeae* isolates from India, Pakistan, and Bhutan in 2007–2011.

**Methods:**

Antimicrobial susceptibility and β-lactamase production were tested for 65 *N*. *gonorrhoeae* isolates from India (n=40), Pakistan (n=18) and Bhutan (n=7) using Etest methodology (eight antimicrobials) and nitrocefin solution, respectively. Resistance determinants, i.e. *penA*, *mtrR*, *porB1b*, *gyrA*, and *parC*, were sequenced. *N*. *gonorrhoeae* multiantigen sequence typing (NG-MAST) was performed for molecular epidemiology.

**Results:**

The highest resistance level was observed for ciprofloxacin (94%), followed by penicillin G (68%), erythromycin (62%), tetracycline (55%), and azithromycin (7.7%). All the isolates were susceptible to ceftriaxone, cefixime, and spectinomycin. Thirty-four (52%) of the isolates were producing β-lactamase. No *penA* mosaic alleles or A501-altered alleles of penicillin-binding protein 2 were identified. Forty-nine NG-MAST STs were identified, of which 42 STs have not been previously described worldwide.

**Conclusions:**

Based on this study, ceftriaxone, cefixime, and spectinomycin can be used as an empirical first-line therapy for gonorrhoea in India, Pakistan, and Bhutan, whereas ciprofloxacin, penicillin G, tetracycline, erythromycin, and azithromycin should not be. It is imperative to strengthen the laboratory infrastructure in this region, as well as to expand the phenotypic and genetic surveillance of antimicrobial resistance, emergence of new resistance, particularly, to extended-spectrum cephalosporins, and molecular epidemiology.

## Background

Gonorrhoea, caused by *Neisseria gonorrhoeae*, is the most common bacterial sexually transmitted infection (STI), with 106.1 million cases among adults estimated in 2008 across the globe [1]. This incidence, according to the World Health Organization (WHO) global estimates, represented a 21% increase since 2005 [1,2]. Accordingly, gonorrhoea is associated with high morbidity and socioeconomic consequences, remaining a major worldwide public health problem [3-5]. During the past 60–70 years, *N*. *gonorrhoeae* has repeatedly demonstrated its enormous ability to develop resistance in a rapid manner to all antimicrobial drugs introduced for first-line therapy, along with a large capacity for the prompt spread of the emerged resistant strains globally [3,4,6-12]. In recent decade in vitro resistance and treatment failures with the currently recommended treatment regimens for gonorrhoea, the extended-spectrum cephalosporins (ESCs) cefixime and ceftriaxone, have been verified in Japan and Europe [13-21]. The threat of widespread resistance to ceftriaxone (the last remaining treatment option for single antimicrobial therapy) and untreatable gonorrhoea is real [6,16,19,22]. In response to this emergent situation the WHO has published its ‘Global Action Plan to Control the Spread and Impact of Antimicrobial Resistance in *Neisseria gonorrhoeae*’ [5,23], and subsequently, the European Centre for Disease Prevention and Control (ECDC) and the Center for Disease Control and Prevention (CDC) in the United States have launched their supplementary regional response plans [24,25].

Mutations in the *penA* gene encoding the penicillin-binding protein 2 (PBP2) is the main determinant for decreased susceptibility and resistance to ESCs. Acquisition of a *penA* mosaic gene or an alteration of amino acid A501 in PBP2 result in increased minimum inhibitory concentrations (MICs) of ESCs [12,26,27]. Furthermore, mutations in the promoter or coding sequence of the repressor gene *mtrR* cause over-expression of the MtrCDE efflux pump system that export the ESCs out from the cell, and alterations of amino acid G101 and A102 in the porin PorB1b (the *penB* resistance determinant), which is encoded by the *porB1b* gene, result in decreased influx of the ESCs. Those mechanisms further decrease the susceptibility to ESCs as well as to other antimicrobials such as penicillins, tetracyclines and macrolides [11,12,28,29]. Resistance to ciprofloxacin is caused by initial mutations in the *gyrA* gene (in particular in codon 91 and 95) that encode an altered subunit GyrA of the enzyme DNA Gyrase, with subsequent mutations in *parC* (encoding an altered subunit ParC of the enzyme DNA Topoisomerase IV) to reach high-level resistance [28,30].

A wide variety of antimicrobials, such as ESCs, but also penicillins, fluoroquinolones, macrolides, tetracycline and spectinomycin, may still be used for the treatment of gonorrhoea in India and Pakistan. Resistance to penicillin, ciprofloxacin, and tetracycline has been reported in India, Sri Lanka, Pakistan, and Bangladesh [31-37]. Further, exceedingly rare resistance (mainly single isolates) to spectinomycin and azithromycin has been observed in India and Bangladesh [32,36,37]. Finally, decreased susceptibility to ESCs has been noted in India and Bangladesh, whereas resistance to ESCs has not yet been reported from this region [31-37]. However, few isolates from this region have been examined and, in most cases, disc diffusion methods that do not reflect the exact MIC have been used for antimicrobial susceptibility testing rather than quality assured, internationally validated methods to determine the exact MIC. Furthermore, genetic antimicrobial resistance determinants, with the exception of determinants for ciprofloxacin resistance [38], have never been studied.

Moreover, it is important to have detailed knowledge about the gonococcal strain populations circulating in different communities, temporal and geographical changes of the populations, and the emergence and transmission patterns of individual strains for the prevention and control of infection. Thus, a highly discriminative, objective, and reproducible characterisation of *N*. *gonorrhoeae* strains can be valuable [39]. Genotypic methods based on DNA sequencing are internationally recommended, of which *N*. *gonorrhoeae* multiantigen sequence typing (NG-MAST) [40] or full- or extended-length *porB* gene sequencing are currently the best methods for fast, objective, portable, highly discriminatory, reproducible, typeable, and high-throughput characterisation [39]. In India and Pakistan gonococcal epidemiology has been explored in a few, mostly outdated studies, using traditional, low discriminatory phenotypic typing methods, such as antimicrobial susceptibility testing, auxotyping, plasmid profiling, and serotyping [41-45]. Only a few minor studies have used genetic gel-based typing methods (e.g., restriction fragment length polymorphism of the whole genomic DNA [46], ribotyping, and Opa-typing [47]). Unfortunately, all these methods suffer from a lack of an objective reading and interpretation of results (compared to sequencing-based methods), international standardisation, and a database for international interlaboratory comparisons [39].

The present study aimed to investigate antimicrobial susceptibility, genetic resistance determinants focussing on extended-spectrum cephalosporins and ciprofloxacin, and the molecular epidemiology of *N*. *gonorrhoeae* strains that were circulating in India, Pakistan, and Bhutan in 2007–2011. Internationally recommended, validated, and quality assured methods were applied for antimicrobial susceptibility testing and molecular epidemiology (NG-MAST).

## Methods

### *N*. *gonorrhoeae* patients and isolates

All viable clinical isolates of *N*. *gonorrhoeae* collected at the VMMC & Safdarjang Hospital, New Delhi, India (n=40; 2007 (3), 2008 (1), 2009 (17), 2010 (19)), Aga Khan University, Karachi, Pakistan (n=18; 2008 (1), 2009 (4), 2010 (3), 2011 (10)), and JDW/NR Hospital, Thimphu, Bhutan (n=7; all from 2010) during 2007–2010 were examined. The isolates were initially cultured from specimens from the urethra (males, n=60) and cervix (females, n=5) of patients presenting with symptoms and signs of gonorrhoea, and verified as *N*. *gonorrhoeae* using growth characteristics on selective culture agar media, Gram staining and oxidase test. All gonococcal isolates were cultured and stored (in −70°C freezer or lyophilized) as an integral part of the routine diagnostics and no personal identification data of any patient was handled. Accordingly, no ethical approval was required. The *N*. *gonorrhoeae* isolates were subsequently transported to the WHO Collaborating Centre for Gonorrhoea and other STIs, Örebro University Hospital, Örebro, Sweden, where the isolates were recultured and verified as *N*. *gonorrhoeae* using growth characteristics on the selective modified Thayer-Martin media, Gram staining, oxidase test and the MicroTrak *N*. *gonorrhoeae* Culture Confirmation Test (Trinity Biotech Plc, Co Wicklow, Ireland). All further testing was performed at the WHO Collaborating Centre for Gonorrhoea and other STIs in Sweden.

The 2008 WHO *N*. *gonorrhoeae* reference strains [30] were used as quality controls in all antimicrobial susceptibility testing and genetic characterisation (see below).

### Antimicrobial susceptibility testing

MIC (mg/L) of all isolates were determined for ceftriaxone, cefixime, penicillin G, tetracycline, erythromycin, azithromycin, ciprofloxacin, and spectinomycin using Etest methodology on Difco GC Medium Base (Becton, Dickinson and Company, Sparks, MD, USA) supplemented with 1% BBL IsoVitaleX Enrichment (Becton, Dickinson and Company, Sparks, MD, USA), according to the instructions from the manufacturer (AB bioMérieux, Solna, Sweden). MIC breakpoints used for determination of susceptibility, intermediate susceptibility, and resistance (Table 1) were largely in accordance with the European Committee on Antimicrobial Susceptibility Testing (EUCAST (http://www.eucast.org); clinical breakpoints, v2.0). Production of β-lactamase was identified by nitrocefin discs (Oxoid, Basingstoke, Hants, England).

**Table 1:** **Antimicrobial susceptibility of 65 *Neisseria gonorrhoeae *isolates from India (n=40), Pakistan (n=18), and Bhutan (n=7) in 2007–2011**

**Antimicrobial**	**Breakpoints (susceptible/resistant, mg/L)**	**MIC range (mg/L)**	**Resistant, number (%)**	**Intermediate susceptible, number (%)**	**Susceptible, number (%)**
Ciprofloxacin	S≤0.03/R>0.06^a^	0.064->32	61 (93.8)	4 (6.2)	0 (0)
Penicillin G^b^	S≤0.06/R>1^a^	0.016->32	44 (67.7)	20 (30.8)	1 (1.5)
Erythromycin^c^	S≤0.25/R>0.5^c^	0.032-128	40 (61.5)	8 (12.3)	17 (26.2)
Tetracycline	S≤0.5/R>1^a^	0.125-64	36 (55.4)	22 (33.8)	7 (10.8)
Azithromycin	S≤0.25/R>0.5^a^	0.016-4	5 (7.7)	10 (15.4)	50 (76.9)
Spectinomycin	S≤64/R>64^a^	4-16	0 (0)	0 (0)	65 (100)
Ceftriaxone	S≤0.12/R>0.12^a^	<0.002-0.064	0 (0)	0 (0)	65 (100)
Cefixime	S≤0.12/R>0.12^a^	<0.016-0.064	0 (0)	0 (0)	65 (100)

^a^Breakpoints according to The European Committee on Antimicrobial Susceptibility Testing. (EUCAST (http://www.eucast.org; Clinical breakpoints v2.0)).

^b^β-lactamase was produced by 34 (52%) of the examined isolates, and all these isolates were considered as resistant to penicillin G independent on their MIC values.

^c^Because of the lack of EUCAST breakpoints, the EUCAST breakpoints for azithromycin were used also for erythromycin.

MIC, minimum inhibitory concentration.

### Isolation of genomic DNA

Genomic DNA was isolated according to the manufacturer’s instructions in a robotised MagNA Pure instrument (Roche, Basel, Switzerland) using the MagNA Pure LC DNA Isolation Kit III.

### Determination of genetic antimicrobial resistance determinants

PCR amplification and sequencing of gonococcal genetic resistance determinants focussing on ESCs and ciprofloxacin, i.e. the *penA*, *mtrR*, *penB*, *gyrA*, and *parC* genes, were performed as described elsewhere [30].

### Molecular epidemiological characterisation

The more variable segments of *porB* (490 bp) and *tbpB* gene (390 bp) examined in NG-MAST [40] were sequenced as described previously [48]. NG-MAST allele numbers and sequence types (STs) were assigned on the NG-MAST website (http://www.ng-mast.net).

## Results

### Antimicrobial susceptibility testing

The results of the antimicrobial susceptibility of the *N*. *gonorrhoeae* isolates (n=65) are summarised in Table 1. Briefly, the resistance was highest to ciprofloxacin (94%), followed by penicillin G (68%), erythromycin (62%), tetracycline (55%), and azithromycin (7.7%). All isolates (100%) were susceptible to ceftriaxone (MIC range: <0.002-0.064 mg/L), cefixime (MIC range: <0.016-0.064 mg/L), and spectinomycin (MIC range: 4–16 mg/L). β-lactamase was produced by 52% (n=34) of the isolates (India: n=22, 55%): Pakistan: n=6, 33%); Bhutan: n=6, 86%)); all those isolates were considered resistant to penicillin G (Table 1).

### Genetic characterisation of *penA* alleles

No *penA* mosaic alleles or A501-altered alleles of PBP2 were identified (Table 2). According to the previously described numbering of PBP2 amino acid sequences in *N*. *gonorrhoeae*[16], PBP2 allele IX was the most prevalent (22 isolates), followed by II (n=19), XIX (n=12), IV (n=7), Modified-XIV (n=3), XII (n=1), and XXXV (n=1). Of these PBP2 alleles, all except PBP2 XXXV contain an insertion of aspartate in amino acid position 345 (D345a), which explains the high level of intermediate susceptibility and resistance to penicillin among the isolates [11,28] (Table 2).

**Table 2:** **Genetic resistance determinants for ceftriaxone, cefixime and ciprofloxacin in *Neisseria gonorrhoeae *isolates from India (n=40), Pakistan (n=18), and Bhutan (n=7) in 2007–2011**

**Antimicrobial (Susceptibility, % of isolates)**	***penA *mosaic allele**	**A501 altered PBP2 allele**	***mtrR *promoter (A-deletion/T-insertion)**	**MtrR coding region (G45D)**	***penB *(alterations in G101/A102)**	***gyrA *(alterations in S91 and D95)**	***parC *(alterations in D86, S87, S88, E91 and A92)**
Ceftriaxone (S, 100%)	None^a^	None^a^	42%/4.6%^b^	11%	D/G (26%), G/S (20%), K/D (18%), K/N (9.2%), N/D (6.2%), K/G (4.6%), G/N (4.6%), N/G (1.5%), G/G (1.5%)	ND	ND
Cefixime (S, 100%)	None^a^	None^a^	42%/4.6%^b^	11%	D/G (26%), G/S (20%), K/D (18%), K/N (9.2%), N/D (6.2%), K/G (4.6%), G/N (4.6%), N/G (1.5%), G/G (1.5%)	ND	ND
Ciprofloxacin (R, 93.8%)	ND	ND	44%/4.9%^b^	11%	D/G (28%), G/S (20%), K/D (15%), K/N (9.8%), N/D (6.6%), K/G (4.9%), G/N (4.9%), N/G (1.6%), G/G (1.6%)	S91F (100%),	D86N (3.3%), S87N (4.9%), S87I (1.6%), S87R (1.6%), E91G (36%), E91K (15%), E91Q (4.9%)
						D95N (46%), D95G (41%), D95A (10%), D95Y (1.6%)	
Ciprofloxacin (I, 6.2%)	ND	ND	None	None	K/D (75%), G/S (25%)	S91F (100%)	None
						D95G (100%)	

S, susceptible; I, intermediate susceptible; R, resistant; ND, not determined because the resistance determinants do not evidently affect this antimicrobial.

^a^The identified penicillin-binding protein 2 (PBP2) alleles were IX (n=22), II (n=19), XIX (n=12), IV (n=7), Modified-XIV (n=3), XII (n=1), and XXXV (n=1). All these PBP2 alleles, with exception of PBP2 XXXV, contain an insertion of aspartate in amino acid position 345 (D345a), which explains the high level of intermediate susceptibility and resistance to penicillin among the isolates [11,28].

^b^One additional isolate (from Pakistan) displayed the recently described C-to-T transition mutation 120 bp upstream of the mtrC start codon, termed *mtr*_120_, that results in a novel consensus −10 element and generation of a novel promoter for *mtrCDE* transcription. This mutation also results in an over-expression of the MtrCDE efflux pump [29].

### Genetic characterisation of resistance determinants that increase the efflux or decrease the influx of antimicrobials

Of the 65 examined isolates, 27 (42%) showed mutations in the inverted repeat region of the *mtrR* promoter (A-deletion (n=24) and T-insertion (n=3)), while G45D amino acid substitution in the DNA-binding motif of MtrR was found in seven (11%) isolates, of which three contained additionally an A-deletion in the *mtrR* promoter region. Furthermore, one isolate from Pakistan displayed the recently described C-to-T transition mutation 120 bp upstream of the mtrC start codon, termed *mtr*_120_, that results in a novel consensus −10 element and generation of a novel promoter for *mtrCDE* transcription. This mutation also results in an over-expression of the MtrCDE efflux pump [29] (Table 2). Of the 15 azithromycin resistant or intermediately susceptible isolates, 11 showed the A-deletion and two a T-insertion in the *mtrR* promoter. Furthermore, *penB* alterations, causing a decreased influx of antimicrobials [12,26,28], were found in 60 of the 65 isolates (92%).

In the absence of *penA* mosaic alleles or A501-altered PBP2 amino acid sequences, isolates containing both the *mtrR* and *penB* resistance determinants still did not have substantially enhanced MICs of the ESCs (ceftriaxone MIC range: 0.002-0.064 mg/L; cefixime MIC range: <0.016-0.064 mg/L), that is, as compared with the remaining isolates (ceftriaxone MIC range: <0.002-0.064 mg/L; cefixime MIC range: <0.016-0.064 mg/L).

### Genetic characterisation of ciprofloxacin resistance determinants

In the present study, *gyrA* ciprofloxacin resistance mutations were found universally: for example, S91F mutations were identified in all 65 (100%) isolates. In addition, *gyrA* D95G mutations were found in 45% of the isolates, D95N in 43%, D95A in 9.2%, and D95Y in 1.5%. Concerning *parC*, A91G mutations were identified in 33.8% of the isolates, followed by A91K in 13.8%, A92Q in 4.6%, S87N in 4.6%, D86N in 3.1%, S82I in 1.5%, and S87R in 1.5% (Table 2).

### Molecular epidemiological characterisation

Among the 65 isolates, 49 NG-MAST STs were identified (Additional file 1: Table S1), of which 42 were novel STs (India: n=27; Pakistan: n=11; Bhutan: n=4). The predominant STs were ST6058 (n=5: Indian isolates), ST6057 (n=4: Indian isolates), and ST6064 (n=3: Bhutan isolates). The remaining STs were represented by only one (39 STs) or two isolates (7 STs).

## Discussion

This study reports the antimicrobial susceptibility/resistance and molecular characteristics of *N*. *gonorrhoeae* isolates from India, Pakistan, and Bhutan in 2007–2011. A high prevalence of resistance was observed for ciprofloxacin (94%), penicillin G (68%), erythromycin (62%), and tetracycline (55%). These data are largely in accordance with previous surveys in the Southeast Asian region. Accordingly, in India, penicillin resistance has varied from 20% to 79%, tetracycline resistance from 0% to 97%, and ciprofloxacin resistance from 11% to 100% [34,49]. In Sri Lanka, 97% and 8.2% resistance have been reported to penicillin and ciprofloxacin, respectively, and in Bangladesh resistance to ciprofloxacin, penicillin, and tetracycline was found to be 76%, 33% and 57%, respectively [34]. Finally, in Pakistan 92%, 87%, and 78% resistance to ofloxacin, penicillin G, and tetracycline, respectively, has been noted [35].

Thus, in the present study none of the isolates was susceptible to ciprofloxacin, with 94% and 6% of the isolates being resistant and intermediately susceptible, respectively. *gyrA* mutations (S91F) were found universally, with many of the isolates containing additional mutations in the quinolone resistance determining regions of the *gyrA* and *parC* genes (Table 2), which confer a high level of resistance to fluoroquinolones (e.g., ciprofloxacin) [28,30,38]. The very high rate of fluoroquinolone resistance may be an indicator of the overuse and misuse of this class of antimicrobials in this region of the world, as caused by over-the-counter availability, unregulated and counterfeit medicines, self-medication or unqualified practitioners who prescribe a full range of treatments [49,50]. Although fluoroquinolones are no longer recommended for first-line treatment of gonorrhoea in most parts of the world [4,11,51-54], most worryingly they are still being used excessively by, in particular, private practitioners and quacks in Southeast Asia.

Another very commonly prescribed antimicrobial in the Southeast Asian region is azithromycin, which is frequently used in syndromic management of STIs because of the convenience of single oral dose therapy for many infections and its efficacy against several STI pathogens, including *N*. *gonorrhoeae*, *Chlamydia trachomatis*, *Mycoplasma genitalium*, *Haemophilus ducreyi*, *Klebsiella granulomatis*, and *Treponema pallidum*[53,55]. Previously, only some single *N*. *gonorrhoeae* isolates with resistance to azithromycin have been reported in India and Bangladesh [36,37]. However, in the present study only 77% of the isolates were susceptible to azithromycin and 23% showed resistance (7.7%) or intermediate susceptibility (15%), showing its unsuitability for use as an empirical first-line therapy for gonorrhoea in this region. Furthermore, resistance to azithromycin, including very high-level resistance, has been described in many countries globally [4,11,12,28,56-60]. Of the 15 isolates that showed resistance or intermediate susceptibility to azithromycin in the present study, 13 (87%) showed mutations in the *mtrR* promoter, which enhances the expression of the MtrCDE efflux pump, exporting azithromycin out from the gonococcal cells and thus confers increased azithromycin MICs in these isolates [11,12,28,29].

Among the isolates examined in this study, no resistance was observed to ceftriaxone, cefixime, and spectinomycin. Accordingly, ceftriaxone, cefixime, and spectinomycin can be recommended as an empirical first-line therapy of gonorrhoea in this region, although judicious use of these antimicrobials (particularly spectinomycin) is imperative. Some single isolates with spectinomycin resistance and strains with “less susceptibility” to ceftriaxone have been reported in India and Bangladesh [32-34,37]. However, few isolates from this region have been examined and mostly disc diffusion methods (with lower breakpoints for “less susceptibility” to ceftriaxone and resistance to spectinomycin compared with international MIC-determining resistance methods) for antimicrobial susceptibility testing have been used rather than internationally validated, quality assured MIC-determining methods. Nevertheless, our data, including MICs of up to 0.064 mg/L for ceftriaxone and cefixime, emphasise the importance of promptly implementing expanded antimicrobial resistance surveillance in India, Pakistan, and Bhutan. Corresponding to the absence of resistance to ESCs, no *penA* mosaic alleles or A501-altered PBP2 amino acid sequences were found in any of the isolates. These alterations of the *penA* gene are critical for resistance to cefixime and ceftriaxone [11-13,16,19].

Disquietingly, a majority of the gonococcal infections (as well as other STIs) in India, Pakistan, and Bhutan remain undiagnosed (using laboratory testing), or go unreported, or both. STIs are also still considered a taboo, and accordingly, people do not visit physicians with their ailment. In other cases the patients have been inadequately treated multiple times when they reach the hospitals, or the samples are inadequately transported to the laboratories, both of which diminish the likelihood of recovering gonococcus in culture. Moreover, most of the laboratories are not well equipped to provide adequate culture and characterisation facilities. Hence, there is a need to strengthen the existing system by providing more resources and training in this region for enhanced surveillance and detection of gonorrhoea and other STIs.

The *N*. *gonorrhoeae* population in India, Pakistan, and Bhutan was found to be highly diversified, with 49 NG-MAST STs identified among the 65 isolates examined (Additional file 1: Table S1). The high number of unique STs (n=39) may be a consequence of random sampling (only viable isolates examined) over several years, sub-optimal diagnostic procedures, incomplete epidemiological surveillance and ineffective contact tracing, local emergence of new STs, and import of strains from abroad. Nevertheless, some minor ST clusters were identified, indicating multiple sexual transmission chains. Furthermore, of the 18 isolates from Pakistan, one isolate was assigned ST368 that also was found in India, suggesting circulation of a few common lineages between the two countries, whereas 11 isolates were of new STs. One isolate from India also belonged to ST1407, which has been shown to be a internationally spread successful gonococcal clone that accounts for the majority of the decreased susceptibility and resistance to ESCs and treatment failures with cefixime worldwide [12,17-19,61,62]. This clone has also shown its ability to develop high-level resistance to ceftriaxone [19]. Surprisingly, the ST1407 isolate from India did not contain the *penA* mosaic allele XXXIV (instead it contained the *penA* allele XXXV) [16] that has been strongly associated with ST1407 [12,17,20,61,63]. Accordingly, despite showing high-level resistance to ciprofloxacin and intermediate susceptibility to azithromycin, the Indian ST1407 isolate displayed low MICs of ceftriaxone (0.008 mg/L) and cefixime (<0.016 mg/L). The seven isolates from Bhutan (belonging to ST6061, ST6062, ST6063, and ST6064) did not share STs with any of the isolates from India or Pakistan.

Because of the small number of isolates examined in the present study, the selection bias for these isolates cultured during several years and the high number of gonococcal infections remaining undiagnosed using laboratory testing in the included countries, the results of the present study need to be interpreted with caution. In future studies, additional isolates will hopefully be available as well as epidemiological data linked to the gonococcal isolates.

## Conclusion

*N*. *gonorrhoeae* strains circulating in India, Pakistan, and Bhutan are genetically highly diverse and exhibit a high resistance to ciprofloxacin, penicillins, erythromycin, and tetracycline. Furthermore, the resistance or intermediate susceptibility to azithromycin was also high. Consequently, cefixime, ceftriaxone, and spectinomycin, which all isolates were susceptible to, are the only antimicrobials that can be recommended for empirical first-line therapy of gonorrhoea in this region. It is essential to strengthen diagnostics capabilities, case reporting, and contact tracing, as well as the surveillance of gonorrhoea and the antimicrobial susceptibility patterns and new emergence of resistance to, in particular, the ESCs (i.e. cefixime and ceftriaxone) in Southeast Asia. Finally, it is also imperative to inform timely and evidence-based update treatment recommendations.

## Competing interests

The authors declare that they no competing interest.

## Authors’ contributions

MU, MB, DG, and SS designed the study, analysed, and interpreted the data. MB, DD, and KJ performed all the initial diagnostics and provided the gonococcal isolates. DG, MI, and SS performed all the laboratory analyses. All authors were involved in the preparation of this paper. All authors read and approved the final manuscript.

## Pre-publication history

The pre-publication history for this paper can be accessed here:

http://www.biomedcentral.com/1471-2334/13/35/prepub

## Supplementary Material

Additional file 1: Table S1 *Neisseria gonorrhoeae* multiantigen sequence typing (NG-MAST) STs of *Neisseria gonorrhoeae* isolates from India (n=40), Pakistan (n=18), and Bhutan (n=7) in 2007–2011. Click here for file
